# Supplementary material for: Thermal Titration Molecular Dynamics (TTMD): Not Your Usual Post-Docking Refinement
Source: Int J Mol Sci. 2023 Feb 10;24(4):3596. doi: 10.3390/ijms24043596 (PMC9968212; doi:10.3390/ijms24043596)
Supplement: Supplementary file 1 [file ijms-24-03596-s001.zip › SMoro_SI_Rev.pdf]

Thermal Titration Molecular Dynamics (TTMD): not your usual post-docking refinement.

Silvia Menin[1§], Matteo Pavan[1§], Veronica Salmaso[1], Mattia Sturlese[1] and Stefano Moro[1*]

[1] Molecular Modeling Section (MMS), Department of Pharmaceutical and Pharmacological Sciences, University of Padova, via Marzolo 5, 35131 Padova, Italy

§ These authors contributed equally

* Correspondence stefano.moro@unipd.it

Supplementary Information

Table S1. Principal chemical properties of the CK1δ ligands utilized in this work. For each ligand, the IC_{50}, the molecular weight, the formal charge, the logP, and the number of hydrogen bond acceptors and donors, are reported.

2D structure	PDB ID	LIG ID	IC_{50} [nM]	MW	Charge	logP	acc H	don H
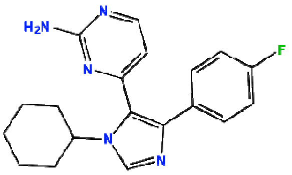	3UZP	0CK	13	337,4	0	2.86	3	1
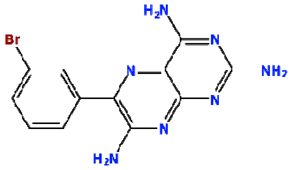	5IH6	AUG	2500	332.17	0	0.22	4	3
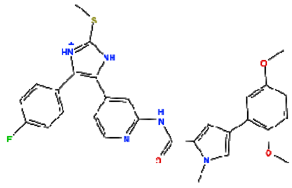	5MQV	D5Q	9	544.63	1	4.72	4	1

Table S2. Results of the TTMD post-docking refinement performed on docking poses generated for each investigated CK1δ protein-ligand complex. For each pose, the representative replicate (MS value nearest to the average) is highlighted in violet.

PDB ID	Pose	MD1	MD2	MD3	MD4	MD5	Mean MS
**3UZP** MS: 0.0024	1	**0.0023**	0.0020	0.0024	0.0019	0.0023	0.0022
	2	**0.0024**	0.0016	0.0021	0.0027	0.0030	0.0024
	3	0.0033	0.0048	0.0077	**0.0034**	0.0033	0.0038
	4	0.0033	**0.0038**	0.0036	0.0066	0.0041	0.0038
	5	0.0031	0.0028	**0.0031**	0.0043	0.0029	0.0030
**5MQV** MS: 0.0030	1	0.0020	0.0026	0.0023	**0.0022**	0.0020	0.0021
	2	0.0025	0.0020	0.0024	**0.0022**	0.0021	0.0022
	3	0.0024	0.0035	**0.0035**	0.0038	0.0035	0.0035
	4	0.0047	**0.0042**	0.0059	0.0041	0.0041	0.0043
	5	0.0034	0.0125	0.0063	0.0048	**0.0056**	0.0055
**5IH6** MS: 0.0052	1	**0.0045**	0.0065	0.0064	0.0036	0.0040	0.0049
	2	0.0028	0.0046	**0.0029**	0.0014	0.0041	0.0032
	3	0.0041	**0.0053**	0.0028	0.0057	0.0100	0.0050
	4	**0.0062**	0.0051	0.0064	0.0061	0.0067	0.0062

	5	0.0061	0.0053	0.0061	0.0052	**0.0057**	0.0057

Table S3. Ligand RMSD (Å) sampled in the last frame of each TTMD simulation performed for the post-docking refinement of poses generated for each investigated CK1δ protein-ligand complex.

PDB ID	Pose	MD1	MD2	MD3	MD4	MD5	Mean RMSD
**3UZP**	1	1.09	0.72	0.68	2.51	1.13	0.98
	2	1.39	0.86	2.35	0.97	1.19	1.18
	3	4.17	6.60	18.28	2.41	2.93	4.57
	4	4.61	2.05	4.99	63.52	3.51	4.37
	5	1.52	3.03	2.40	1.89	1.51	1.94
**5MQV**	1	2.07	3.30	1.72	1.75	2.25	2.02
	2	3.49	5.80	2.48	2.91	2.51	2.97
	3	2.98	3.69	2.37	3.03	3.21	3.07
	4	5.27	4.58	14.52	4.75	8.24	6.09
	5	4.18	12.11	20.34	4.08	9.56	8.62
**5IH6**	1	4.75	50.34	6.79	50.55	26.02	27.72
	2	6.51	6.29	3.93	1.88	2.30	4.17
	3	5.55	4.88	6.00	10.81	6.49	6.01
	4	5.00	8.80	8.52	3.86	26.17	7.44
	5	3.46	6.34	7.90	10.58	2.66	5.90

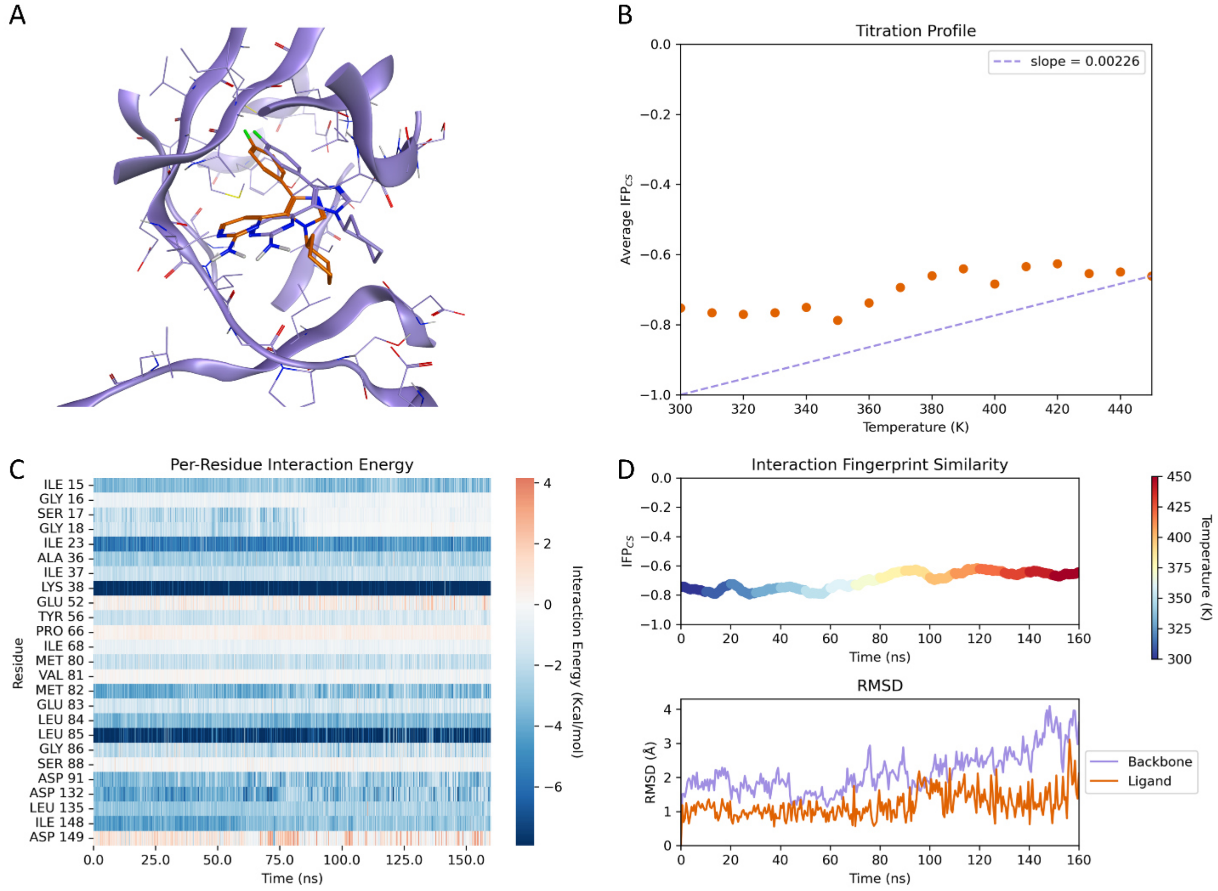

Figure S1. Analyses performed on a representative TTMD trajectory (**MD1**) for the docking pose of ligand **0CK** (PDB ID: **3UZP**) associated with the lowest MS score (**pose 1**). A) superposition between the ligand conformation sampled in the last trajectory frame (orange) and the reference ligand binding mode (violet). B) titration profile: the average IFPCS value (adimensional units) for each "TTMD step" is reported as a function of the step temperature (K) in the form of orange dots. A violet straight line connects the start and the final point of the TTMD simulation, with the slope of the straightline being reported in the legend. C) time-dependent per-residue decomposition of the protein-ligand interaction energy, defined as a sum of the van der Waals and electrostatic contribution. The 25 most contacted residues alongside theTTMD trajectory are reported on the vertical axis, while the simulation time (ns) is reported on the horizontal axis. D) upper: time-dependent evolution of the IFPCS score; lower: time-dependent evolution of the RMSD for both the ligand(orange) and the protein backbone (violet) throughout the TTMD simulation. In both plots, the simulation time (ns) is reported on the horizontal axis, while the IFPCS (adimensional units) and RMSD (Å) value, respectively, are reported on the vertical axis.

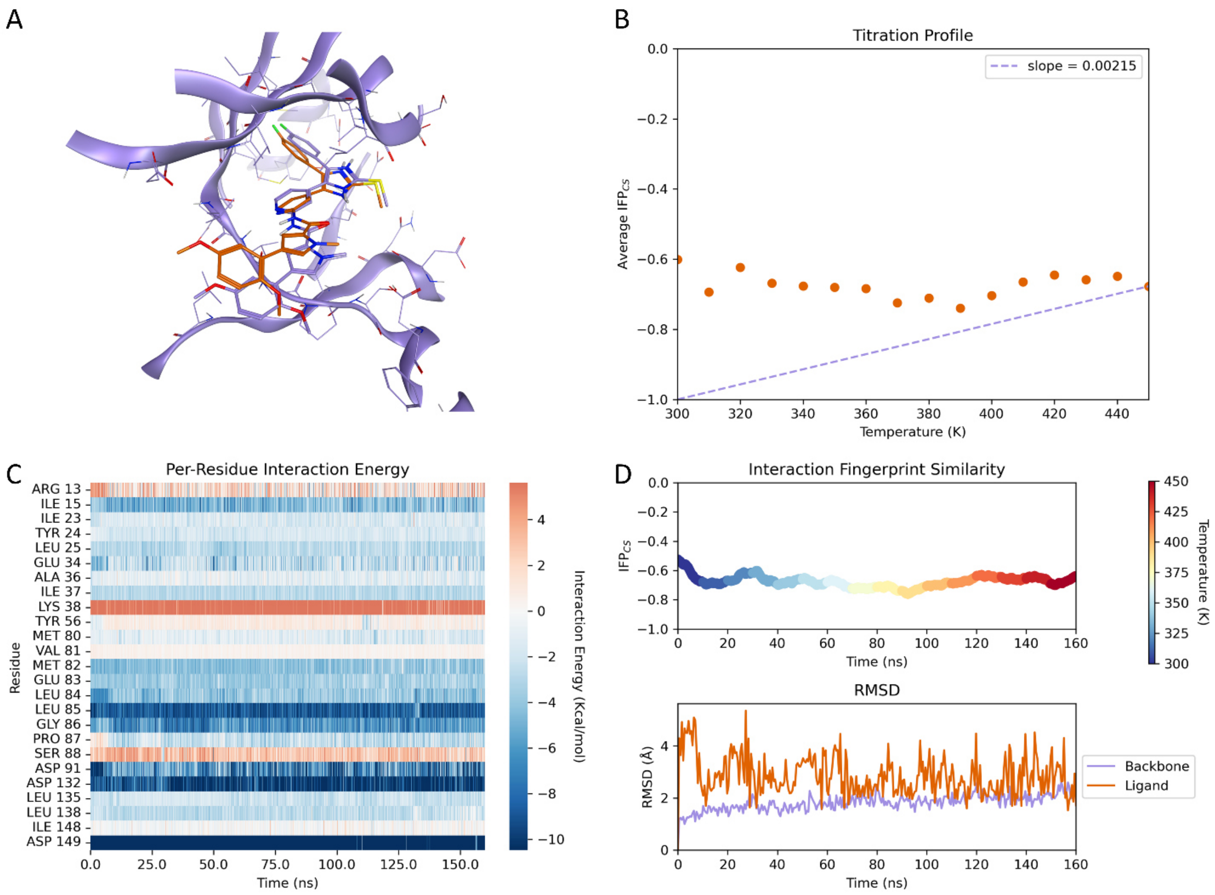

Figure S2. Analyses performed on a representative TTMD trajectory (**MD4**) for the docking pose of ligand **D5Q** (PDB ID: **5MQV**) associated with the lowest MS score (**pose 1**). A) superposition between the ligand conformation sampled in the last trajectory frame (orange) and the reference ligand binding mode (violet). B) titration profile: the average IFPcs value (adimensional units) for each "TTMD step" is reported as a function of the step temperature (K) in the form of orange dots. A violet straight line connects the start and the final point of the TTMD simulation, with the slope of the straight line being reported in the legend. C) time-dependent per-residue decomposition of the protein-ligand interaction energy, defined as a sum of the van der Waals and electrostatic contribution. The 25 most contacted residues alongside the TTMD trajectory are reported on the vertical axis, while the simulation time (ns) is reported on the horizontal axis. D) upper: time-dependent evolution of the IFPcs score; lower: time-dependent evolution of the RMSD for both the ligand (orange) and the protein backbone (violet) throughout the TTMD simulation. In both plots, the simulation time (ns) is reported on the horizontal axis, while the IFPcs (adimensional units) and RMSD (Å) value, respectively, are reported on the vertical axis.

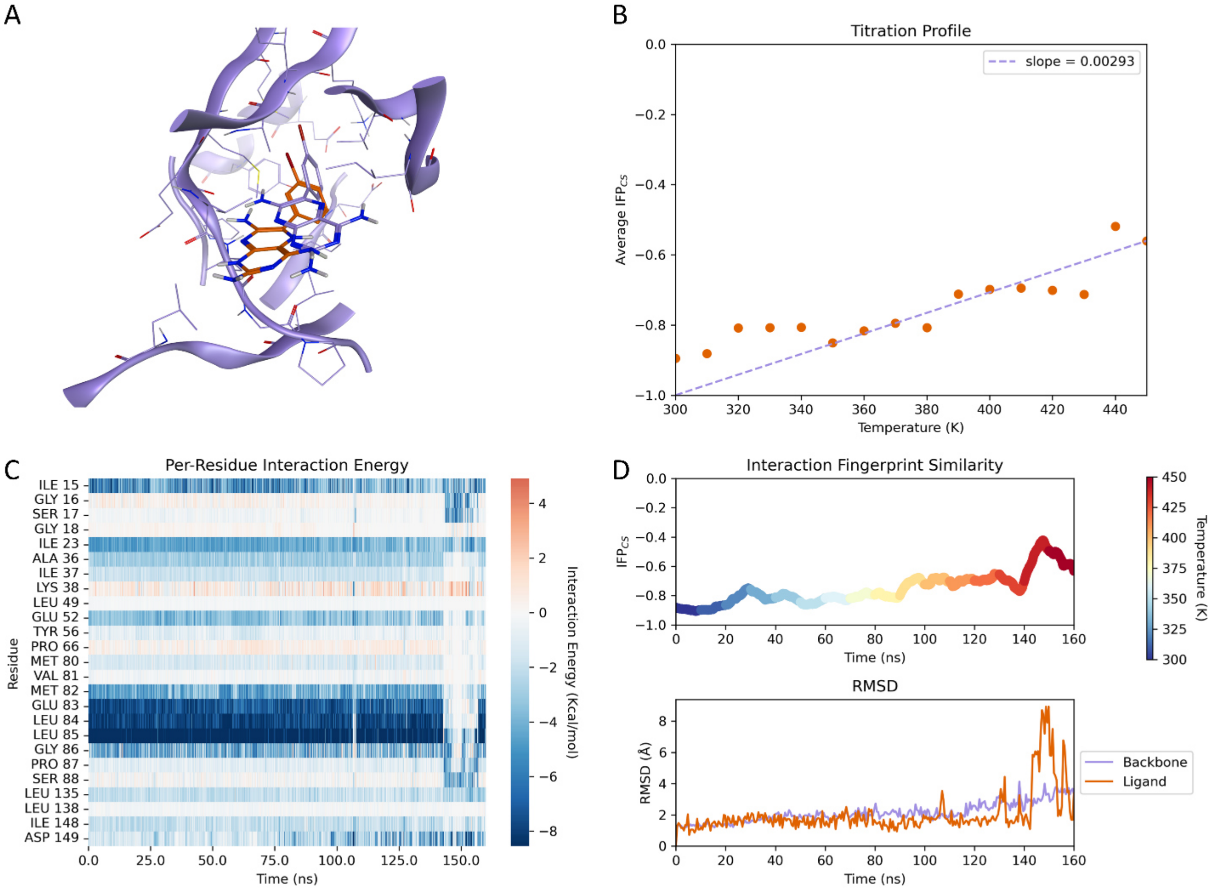

Figure S3. Analyses performed on a representative TTMD trajectory (**MD3**) for the docking pose of ligand **AUG** (PDB ID: **5IH6**) associated with the lowest MS score (**pose 2**). A) superposition between the ligand conformation sampled in the last trajectory frame (orange) and the reference ligand binding mode (violet). B) titration profile: the average IFPcs value (adimensional units) for each "TTMD step" is reported as a function of the step temperature (K) in the form of orange dots. A violet straight line connects the start and the final point of the TTMD simulation, with the slope of the straight line being reported in the legend. C) time-dependent per-residue decomposition of the protein-ligand interaction energy, defined as a sum of the van der Waals and electrostatic contribution. The 25 most contacted residues alongside the TTMD trajectory are reported on the vertical axis, while the simulation time (ns) is reported on the horizontal axis. D) upper: time-dependent evolution of the IFPcs score; lower: time-dependent evolution of the RMSD for both the ligand (orange) and the protein backbone (violet) throughout the TTMD simulation. In both plots, the simulation time (ns) is reported on the horizontal axis, while the IFPcs (adimensional units) and RMSD (Å) value, respectively, are reported on the vertical axis.

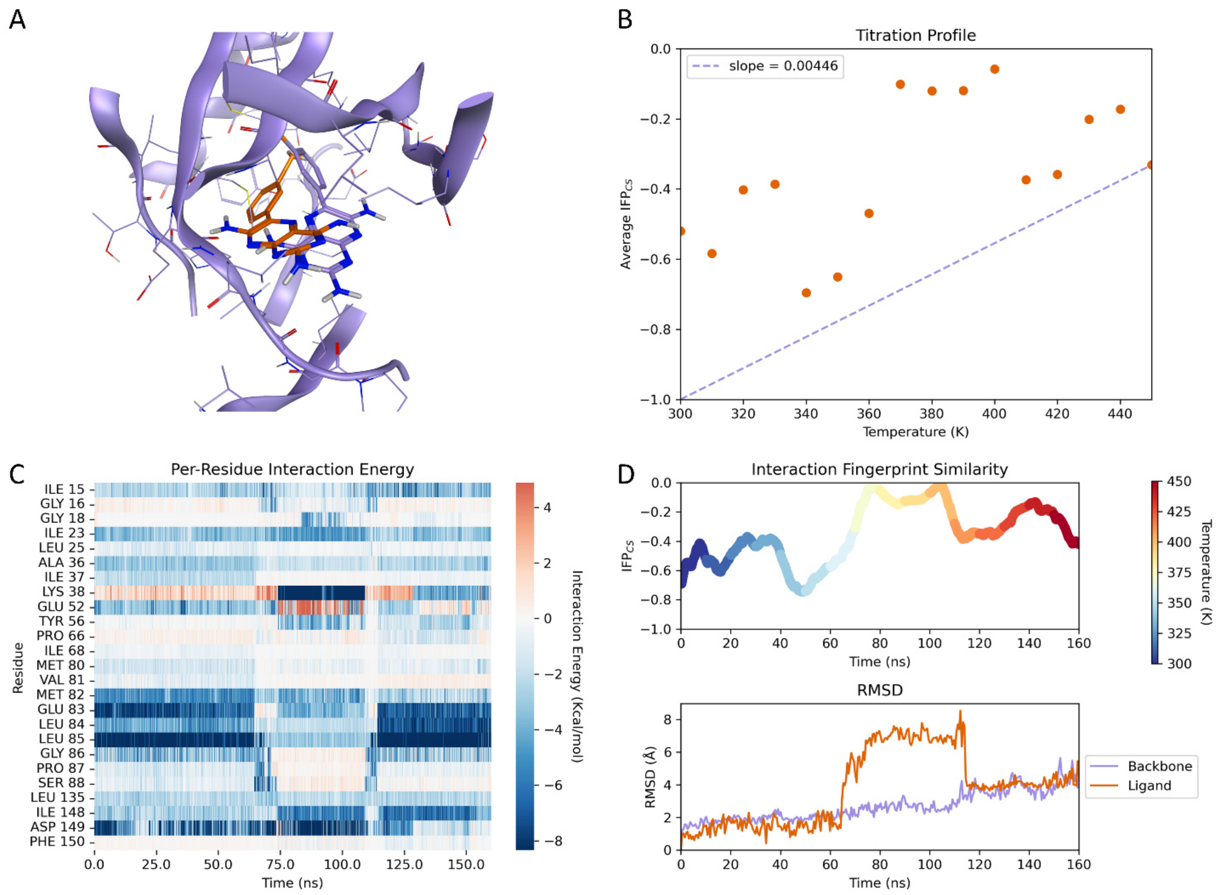

Figure S4. Analyses performed on a representative TTMD trajectory (**MD1**) for the docking pose of ligand **AUG** (PDB ID: **5IH6**) associated with the lowest RMSD to the reference (**pose 1**). A) superposition between the ligand conformation sampled in the last trajectory frame (orange) and the reference ligand binding mode (violet). B) titration profile: the average IFP_{CS} value (adimensional units) for each "TTMD step" is reported as a function of the step temperature (K) in the form of orange dots. A violet straight line connects the start and the final point of the TTMD simulation, with the slope of the straight line being reported in the legend. C) time-dependent per-residue decomposition of the protein-ligand interaction energy, defined as a sum of the van der Waals and electrostatic contribution. The 25 most contacted residues alongside the TTMD trajectory are reported on the vertical axis, while the simulation time (ns) is reported on the horizontal axis. D) upper: time-dependent evolution of the IFP_{CS} score; lower: time-dependent evolution of the RMSD for both the ligand (orange) and the protein backbone (violet) throughout the TTMD simulation. In both plots, the simulation time (ns) is reported on the horizontal axis, while the IFP_{CS} (adimensional units) and RMSD (Å) value, respectively, are reported on the vertical axis.

Table S4. Principal chemical properties of the CK2 ligands utilized in this work. For each ligand, the K_d, the molecular weight, the formal charge, the logP, and the number of hydrogen bond acceptors and donors are reported.

2D structure	PDB ID	LIG ID	K_d [nM]	MW	Charge	logP	acc H	don H
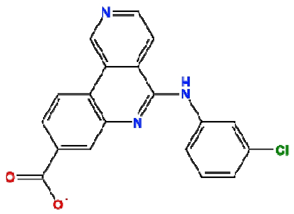	3PE1	3NG	1.5	348.77	-1	4.12	2	1
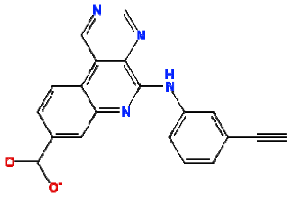	3PE2	E1B	2.3	339.33	-1	3.33	3	1
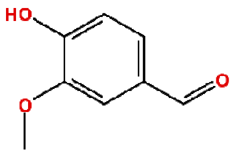	6HOU	V55	53400	152.15	0	1.25	3	1

Table S5. Results of the TTMD post-docking refinement performed on docking poses generated for each investigated CK2 protein-ligand complex. For each pose, the representative replicate (MS value nearest to the average) is highlighted in violet.

PDB ID	Pose	MD1	MD2	MD3	MD4	MD5	Mean MS
**3PE1**<br>MS: 0.0022	1	0.0015	0.0018	**0.0021**	0.0024	0.0022	0.0020
	2	0.0024	0.0033	**0.0026**	0.0031	0.0023	0.0027
	3	0.0052	0.0071	**0.0044**	0.0037	0.0031	0.0044
	4	0.0083	0.0038	0.0052	0.0040	**0.0052**	0.0048
	5	0.0034	0.0036	0.0035	**0.0034**	0.0028	0.0034
**3PE2**<br>MS: 0.0017	1	**0.0020**	0.0015	0.0022	0.0019	0.0027	0.0020
	2	0.0020	**0.0021**	0.0022	0.0027	0.0017	0.0021
	3	**0.0046**	0.0062	0.0036	0.0038	0.0091	0.0049
	4	0.0020	0.0042	0.0066	**0.0021**	0.0018	0.0027
	5	0.0030	0.0026	0.0039	0.0023	**0.0027**	0.0027
**6HOU**<br>MS: 0.0057	1	**0.0167**	0.0143	0.1000	**0.0167**	0.0067	0.0159
	2	0.0250	**0.0167**	0.0111	**0.0167**	0.0054	0.0148
	3	**0.0100**	0.0083	0.0250	0.0067	0.0250	0.0144
	4	0.0500	**0.0125**	0.0250	0.0077	0.0091	0.0155
	5	0.0021	0.0167	**0.0111**	0.0125	0.0100	0.0112

Table S6. Ligand RMSD (Å) sampled in the last frame of each TTMD simulation performed for the post-docking refinement of poses generated for each investigated CK2 protein-ligand complex.

PDB ID	Pose	MD1	MD2	MD3	MD4	MD5	Mean RMSD
**3PE1**	1	2.47	2.53	1.49	3.38	3.94	2.79
	2	2.03	1.86	5.08	3.08	1.38	2.32
	3	3.81	16.29	5.99	8.84	3.04	6.21
	4	17.63	4.06	6.40	4.42	5.20	5.34
	5	6.08	6.56	6.44	6.86	5.97	6.36
**3PE2**	1	1.52	2.29	3.82	2.13	7.72	2.75
	2	2.59	3.27	2.60	4.13	3.44	3.10
	3	8.78	15.82	8.17	8.31	13.63	10.24
	4	3.86	7.66	11.61	3.82	3.13	5.11
	5	3.23	3.05	9.09	8.78	4.43	5.48
**6HOU**	1	54.37	50.78	10.46	33.76	33.74	39.43
	2	12.55	11.60	71.80	50.57	13.14	25.42
	3	18.81	44.92	16.82	49.04	35.09	32.94
	4	12.03	42.73	39.30	29.65	14.09	27.68
	5	4.29	10.96	42.14	41.11	20.99	24.35

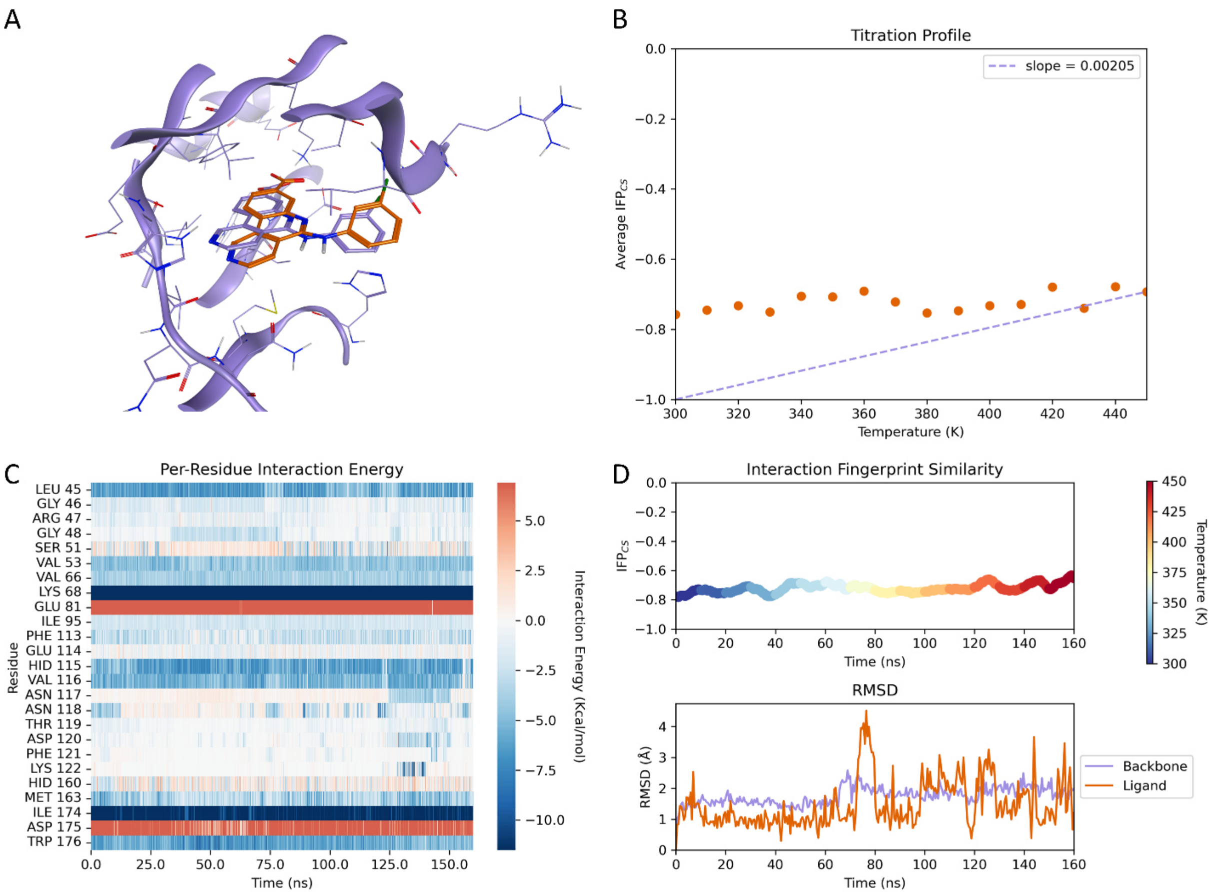

Figure S5. Analyses performed on a representative TTMD trajectory (**MD3**) for the docking pose of ligand **3NG** (PDB ID: **3PE1**) associated with the lowest MS score (**pose 1**). A) superposition between the ligand conformation sampled in the last trajectory frame (orange) and the reference ligand binding mode (violet). B) titration profile: the average IFPcs value (adimensional units) for each "TTMD step" is reported as a function of the step temperature (K) in the form of orange dots. A violet straight line connects the start and the final point of the TTMD simulation, with the slope of the straight line being reported in the legend. C) time-dependent per-residue decomposition of the protein-ligand interaction energy, defined as a sum of the van der Waals and electrostatic contribution. The 25 most contacted residues alongside the TTMD trajectory are reported on the vertical axis, while the simulation time (ns) is reported on the horizontal axis. D) upper: time-dependent evolution of the IFPcs score; lower: time-dependent evolution of the RMSD for both the ligand (orange) and the protein backbone (violet) throughout the TTMD simulation. In both plots, the simulation time (ns) is reported on the horizontal axis, while the IFPcs (adimensional units) and RMSD (Å) value, respectively, are reported on the vertical axis.

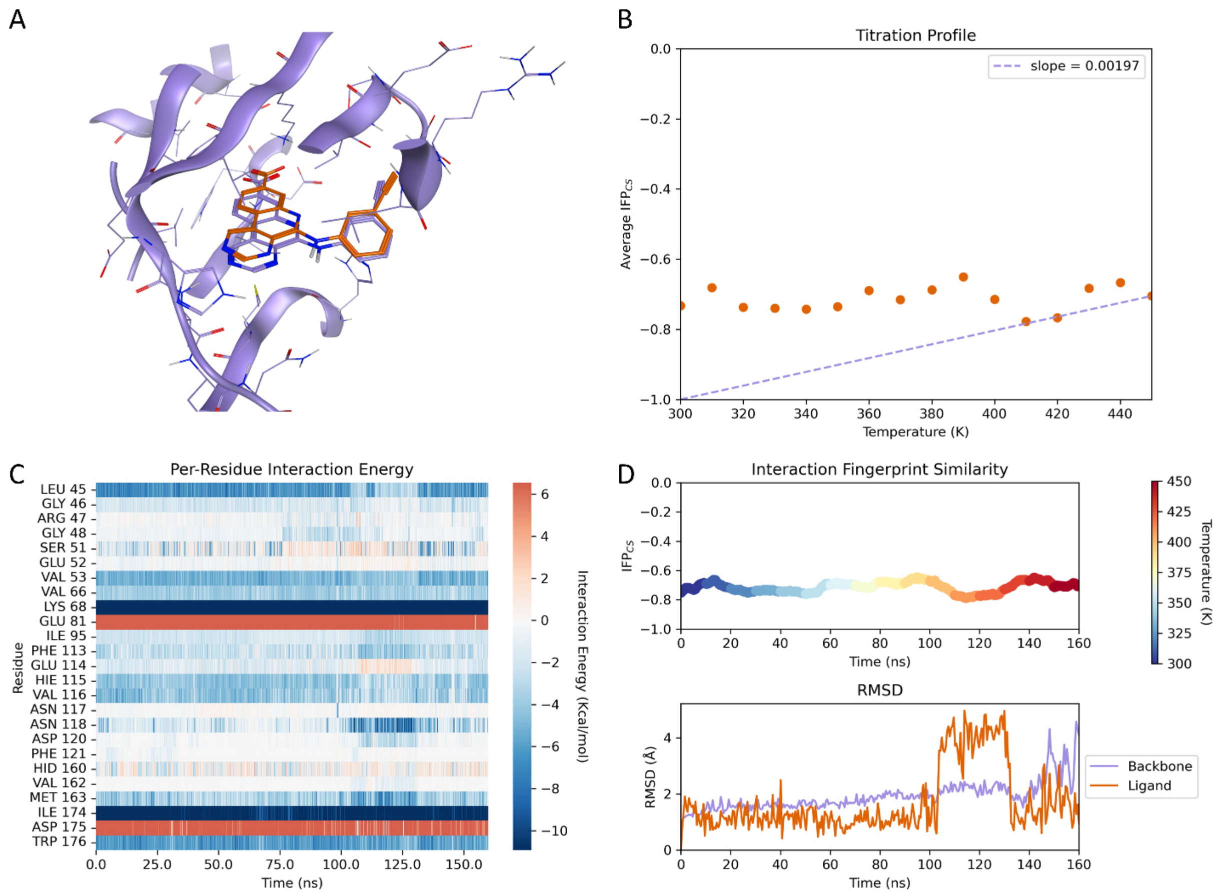

Figure S6. Analyses performed on a representative TTMD trajectory (**MD1**) for the docking pose of ligand **E1B** (PDB ID: **3PE2**) associated with the lowest MS score (**pose 1**). A) superposition between the ligand conformation sampled in the last trajectory frame (orange) and the reference ligand binding mode (violet). B) titration profile: the average IFPcs value (adimensional units) for each "TTMD step" is reported as a function of the step temperature (K) in the form of orange dots. A violet straight line connects the start and the final point of the TTMD simulation, with the slope of the straight line being reported in the legend. C) time-dependent per-residue decomposition of the protein-ligand interaction energy, defined as a sum of the van der Waals and electrostatic contribution. The 25 most contacted residues alongside the TTMD trajectory are reported on the vertical axis, while the simulation time (ns) is reported on the horizontal axis. D) upper: time-dependent evolution of the IFPcs score; lower: time-dependent evolution of the RMSD for both the ligand (orange) and the protein backbone (violet) throughout the TTMD simulation. In both plots, the simulation time (ns) is reported on the horizontal axis, while the IFPcs (adimensional units) and RMSD (Å) value, respectively, are reported on the vertical axis.

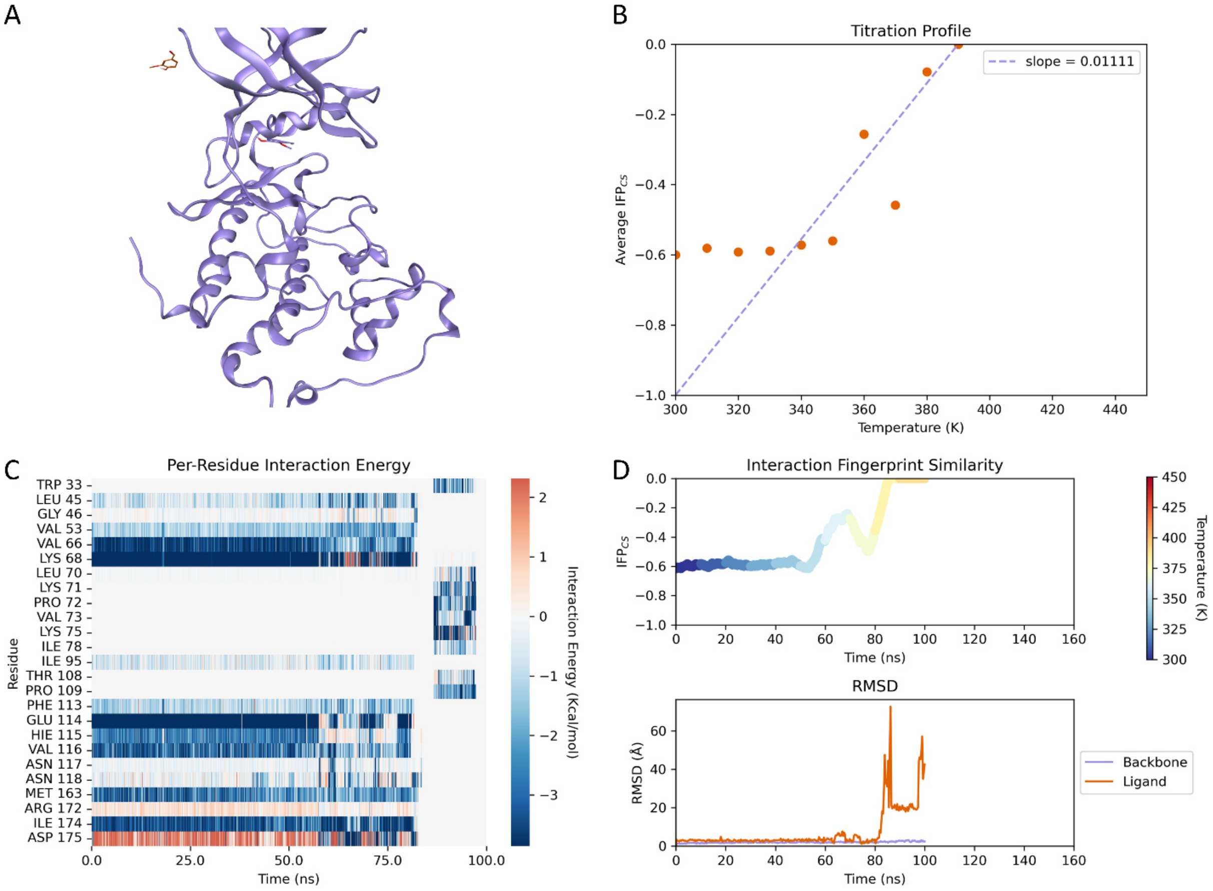

Figure S7. Analyses performed on a representative TTMD trajectory (**MD3**) for the docking pose of ligand **V55** (PDB ID: **6HOU**) associated with the lowest MS score (**pose 5**). A) superposition between the ligand conformation sampled in the last trajectory frame (orange) and the reference ligand binding mode (violet). B) titration profile: the average IFPcs value (adimensional units) for each "TTMD step" is reported as a function of the step temperature (K) in the form of orange dots. A violet straight line connects the start and the final point of the TTMD simulation, with the slope of the straight line being reported in the legend. C) time-dependent per-residue decomposition of the protein-ligand interaction energy, defined as a sum of the van der Waals and electrostatic contribution. The 25 most contacted residues alongside the TTMD trajectory are reported on the vertical axis, while the simulation time (ns) is reported on the horizontal axis. D) upper: time-dependent evolution of the IFPcs score; lower: time-dependent evolution of the RMSD for both the ligand (orange) and the protein backbone (violet) throughout the TTMD simulation. In both plots, the simulation time (ns) is reported on the horizontal axis, while the IFPcs (adimensional units) and RMSD (Å) value, respectively, are reported on the vertical axis.

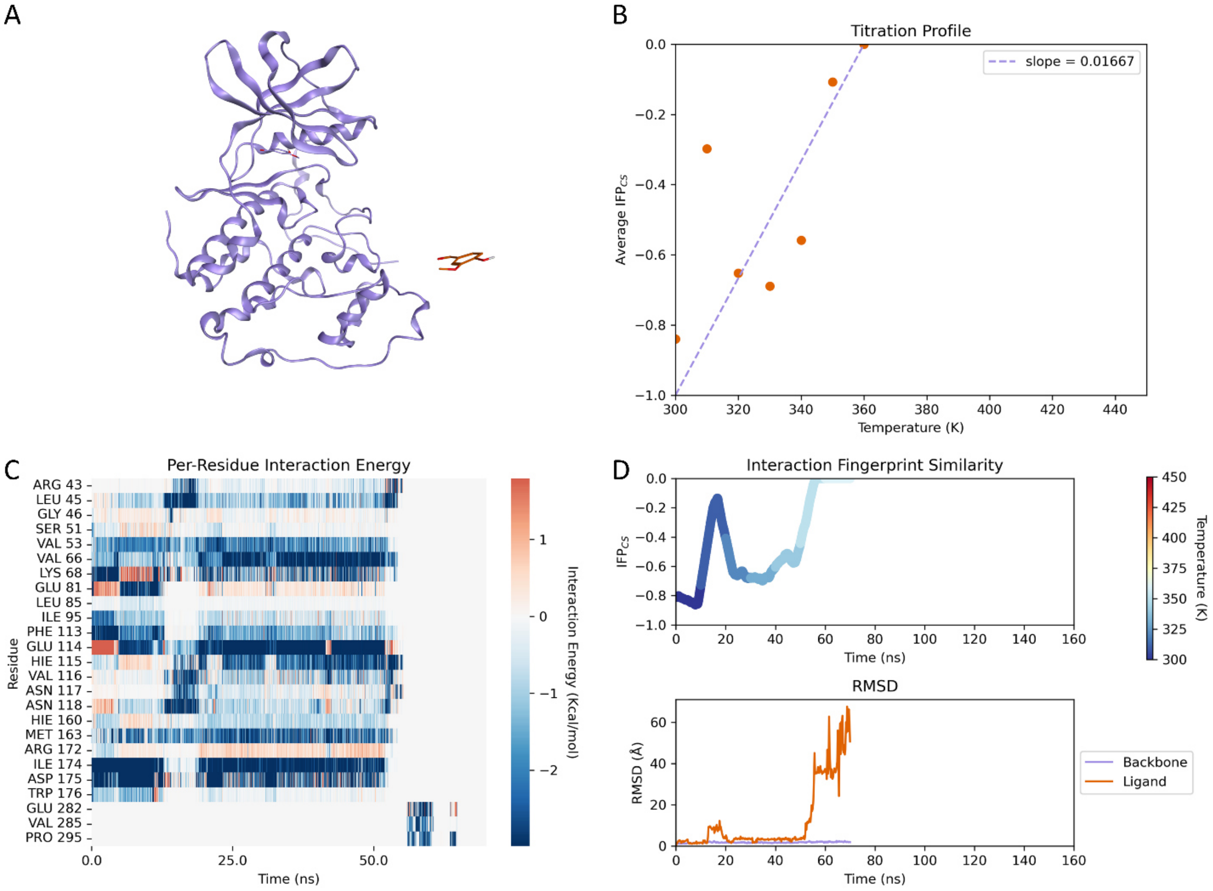

Figure S8. Analyses performed on a representative TTMD trajectory (**MD4**) for the docking pose of ligand **V55** (PDB ID: **6HOU**) associated with the lowest RMSD to the reference (**pose 2**). A) superposition between the ligand conformation sampled in the last trajectory frame (orange) and the reference ligand binding mode (violet). B) titration profile: the average IFPcs value (adimensional units) for each "TTMD step" is reported as a function of the step temperature (K) in the form of orange dots. A violet straight line connects the start and the final point of the TTMD simulation, with the slope of the straight line being reported in the legend. C) time-dependent per-residue decomposition of the protein-ligand interaction energy, defined as a sum of the van der Waals and electrostatic contribution. The 25 most contacted residues alongside the TTMD trajectory are reported on the vertical axis, while the simulation time (ns) is reported on the horizontal axis. D) upper: time-dependent evolution of the IFPcs score; lower: time-dependent evolution of the RMSD for both the ligand (orange) and the protein backbone (violet) throughout the TTMD simulation. In both plots, the simulation time (ns) is reported on the horizontal axis, while the IFPcs (adimensional units) and RMSD (Å) value, respectively, are reported on the vertical axis.

Table S7. Principal chemical properties of the PDK2 ligands utilized in this work. For each ligand, the K_d, the molecular weight, the formal charge, the logP, and the number of hydrogen bond acceptors and donors are reported.

2D structure	PDB ID	LIG ID	K_d [nM]	MW	Charge	logP	acc H	don H
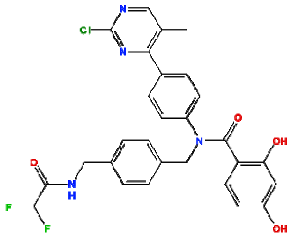	4V25	SZ6	150	552.97	0	5.91	6	3
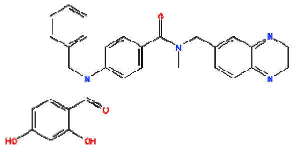	5M4M	7FW	1	518.57	0	6.04	6	2
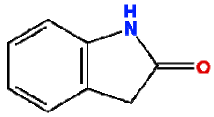	7EA0	W6P	958000	133.15	0	1.18	1	1

Table S8. Results of the TTMD post-docking refinement performed on docking poses generated for each investigated PDK2 protein-ligand complex. For each pose, the representative replicate (MS value nearest to the average) is highlighted in violet.

PDB ID	Pose	MD1	MD2	MD3	MD4	MD5	Mean MS
**4V25** MS: 0.0023	1	**0.0031**	0.0024	0.0024	0.0032	0.0049	0.0029
	2	**0.0034**	0.0067	0.0032	0.0041	0.0029	0.0036
	3	0.0071	0.0042	**0.0060**	0.0049	0.0063	0.0057
	4	0.0064	**0.0066**	0.0111	0.0500	0.0058	0.0080
	5	0.0035	0.0034	0.0039	0.0039	**0.0037**	0.0037
**5M4M** MS: 0.0037	1	0.0030	**0.0028**	0.0025	0.0026	0.0032	0.0028
	2	0.0067	0.0052	0.0067	**0.0057**	0.0041	0.0059
	3	0.0049	**0.0052**	0.0054	0.0055	0.0052	0.0052
	4	0.0029	0.0037	0.0029	**0.0030**	0.0031	0.0030
	5	**0.0036**	0.0035	0.0042	0.0039	0.0032	0.0036
**7EA0** MS: 0.0070	1	0.0030	0.0083	0.0066	**0.0071**	0.0091	0.0073
	2	0.0091	**0.0071**	0.0143	0.0036	0.0050	0.0071
	3	0.0250	0.0047	0.0111	0.0021	**0.0083**	0.0080
	4	0.0067	**0.0091**	0.0038	0.0167	0.0100	0.0086
	5	**0.0071**	0.0049	0.0067	0.0111	0.0091	0.0076

Table S9. Ligand RMSD (Å) sampled in the last frame of each TTMD simulation performed for the post-docking refinement of poses generated for each investigated PDK2 protein-ligand complex.

PDB ID	Pose	MD1	MD2	MD3	MD4	MD5	Mean RMSD
**4V25**	1	2.48	2.46	2.37	2.37	1.48	2.40
	2	8.44	38.67	5.69	5.50	7.12	7.08
	3	24.58	8.96	6.74	7.45	14.42	10.28
	4	14.99	8.13	21.78	17.46	9.15	13.87
	5	5.43	5.13	6.92	5.07	4.71	5.21
**5M4M**	1	2.48	3.15	2.58	2.17	1.43	2.41
	2	40.21	11.38	25.42	10.34	7.57	15.71
	3	11.03	7.43	7.88	11.04	8.34	9.08
	4	4.20	4.55	4.67	4.46	5.52	4.56
	5	6.02	4.72	9.71	4.75	3.16	5.16
**7EA0**	1	2.43	79.12	9.34	18.83	35.60	21.26
	2	55.46	73.99	8.53	1.90	5.86	23.28
	3	18.73	2.57	50.95	3.72	74.47	24.47
	4	18.57	26.67	1.61	78.54	43.92	29.72
	5	13.69	1.10	43.22	49.24	18.80	25.24

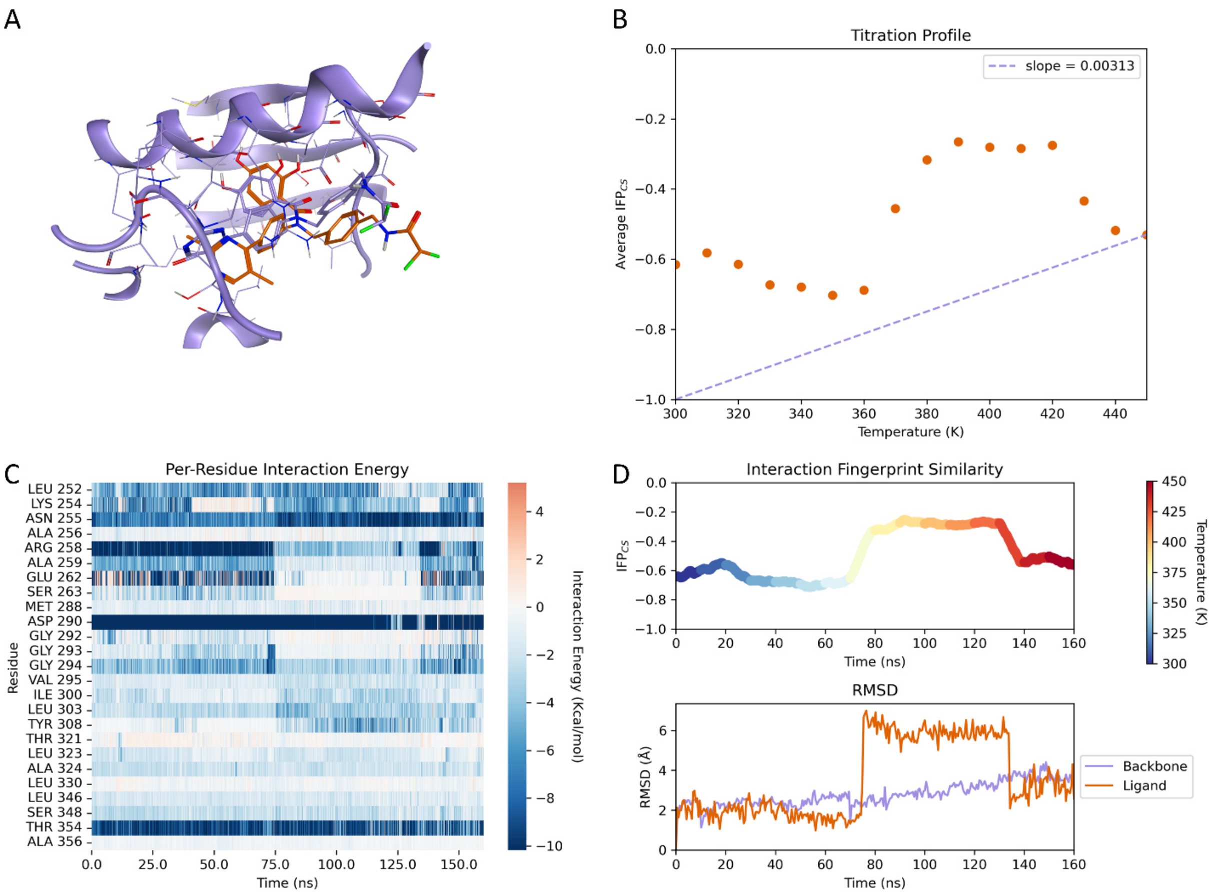

Figure S9. Analyses performed on a representative TTMD trajectory (**MD1**) for the docking pose of ligand **SZ6** (PDB ID: **4V25**) associated with the lowest MS score (**pose 1**). A) superposition between the ligand conformation sampled in the last trajectory frame (orange) and the reference ligand binding mode (violet). B) titration profile: the average IFPcs value (adimensional units) for each "TTMD step" is reported as a function of the step temperature (K) in the form of orange dots. A violet straight line connects the start and the final point of the TTMD simulation, with the slope of the straight line being reported in the legend. C) time-dependent per-residue decomposition of the protein-ligand interaction energy, defined as a sum of the van der Waals and electrostatic contribution. The 25 most contacted residues alongside the TTMD trajectory are reported on the vertical axis, while the simulation time (ns) is reported on the horizontal axis. D) upper: time-dependent evolution of the IFPcs score; lower: time-dependent evolution of the RMSD for both the ligand (orange) and the protein backbone (violet) throughout the TTMD simulation. In both plots, the simulation time (ns) is reported on the horizontal axis, while the IFPcs (adimensional units) and RMSD (Å) value, respectively, are reported on the vertical axis.

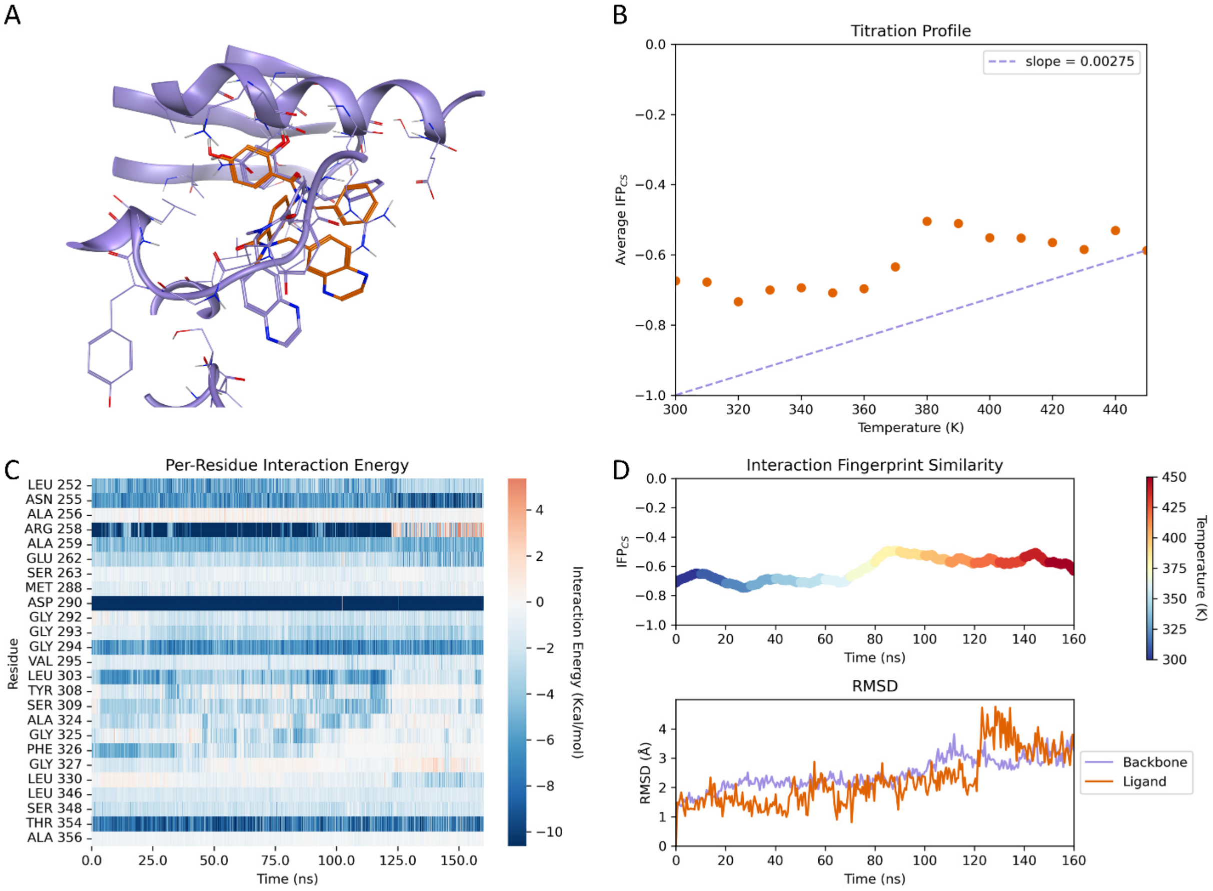

Figure S10. Analyses performed on a representative TTMD trajectory (**MD2**) for the docking pose of ligand **7FW** (PDB ID: **5M4M**) associated with the lowest MS score (**pose 1**). A) superposition between the ligand conformation sampled in the last trajectory frame (orange) and the reference ligand binding mode (violet). B) titration profile: the average IFPcs value (adimensional units) for each "TTMD step" is reported as a function of the step temperature (K) in the form of orange dots. A violet straight line connects the start and the final point of the TTMD simulation, with the slope of the straight line being reported in the legend. C) time-dependent per-residue decomposition of the protein-ligand interaction energy, defined as a sum of the van der Waals and electrostatic contribution. The 25 most contacted residues alongside the TTMD trajectory are reported on the vertical axis, while the simulation time (ns) is reported on the horizontal axis. D) upper: time-dependent evolution of the IFPcs score; lower: time-dependent evolution of the RMSD for both the ligand (orange) and the protein backbone (violet) throughout the TTMD simulation. In both plots, the simulation time (ns) is reported on the horizontal axis, while the IFPcs (adimensional units) and RMSD (Å) value, respectively, are reported on the vertical axis.

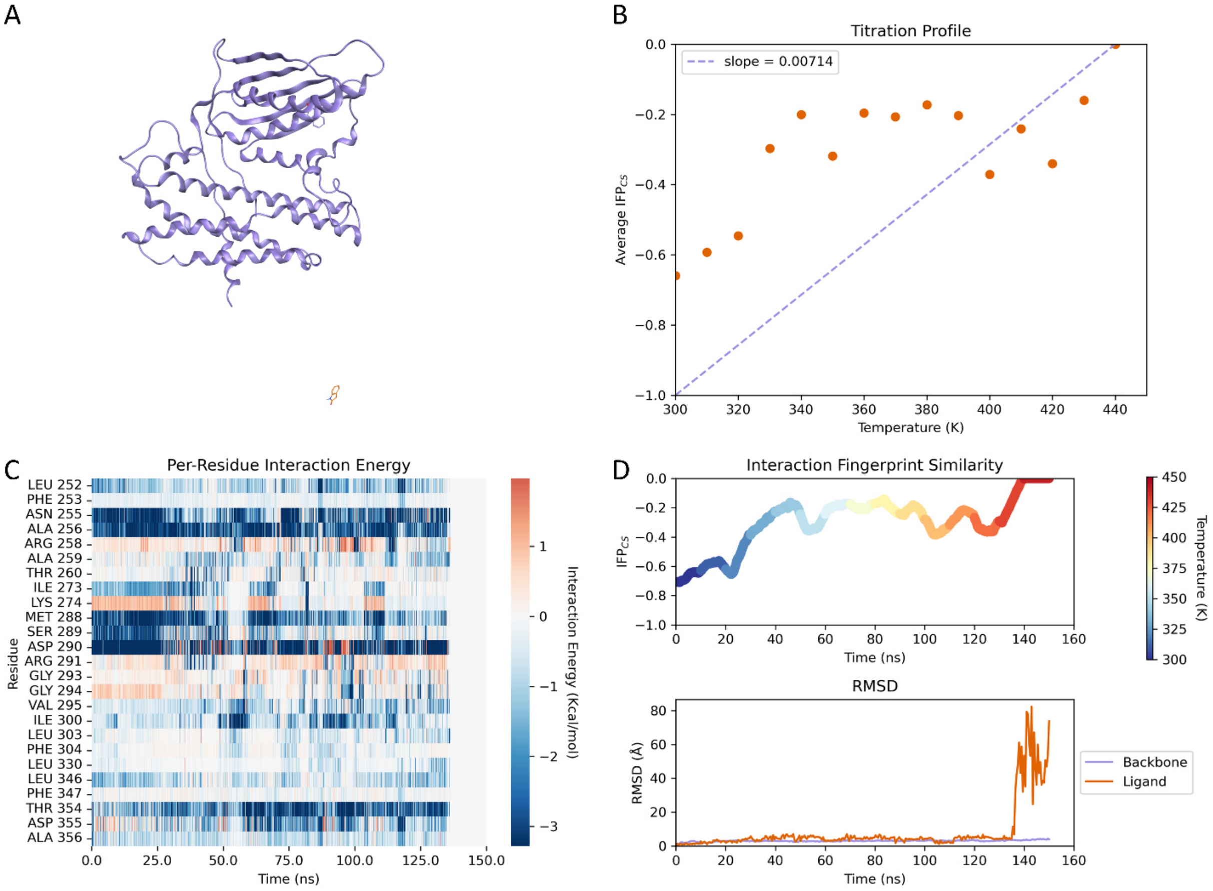

Figure S11. Analyses performed on a representative TTMD trajectory (**MD2**) for the docking pose of ligand **W6P** (PDB ID: **7EA0**) associated with the lowest MS score (**pose 2**). A) superposition between the ligand conformation sampled in the last trajectory frame (orange) and the reference ligand binding mode (violet). B) titration profile: the average IFP$_{CS}$ value (adimensional units) for each "TTMD step" is reported as a function of the step temperature (K) in the form of orange dots. A violet straight line connects the start and the final point of the TTMD simulation, with the slope of the straight line being reported in the legend. C) time-dependent per-residue decomposition of the protein-ligand interaction energy, defined as a sum of the van der Waals and electrostatic contribution. The 25 most contacted residues alongside the TTMD trajectory are reported on the vertical axis, while the simulation time (ns) is reported on the horizontal axis. D) upper: time-dependent evolution of the IFP$_{CS}$ score; lower: time-dependent evolution of the RMSD for both the ligand (orange) and the protein backbone (violet) throughout the TTMD simulation. In both plots, the simulation time (ns) is reported on the horizontal axis, while the IFP$_{CS}$ (adimensional units) and RMSD (Å) value, respectively, are reported on the vertical axis.

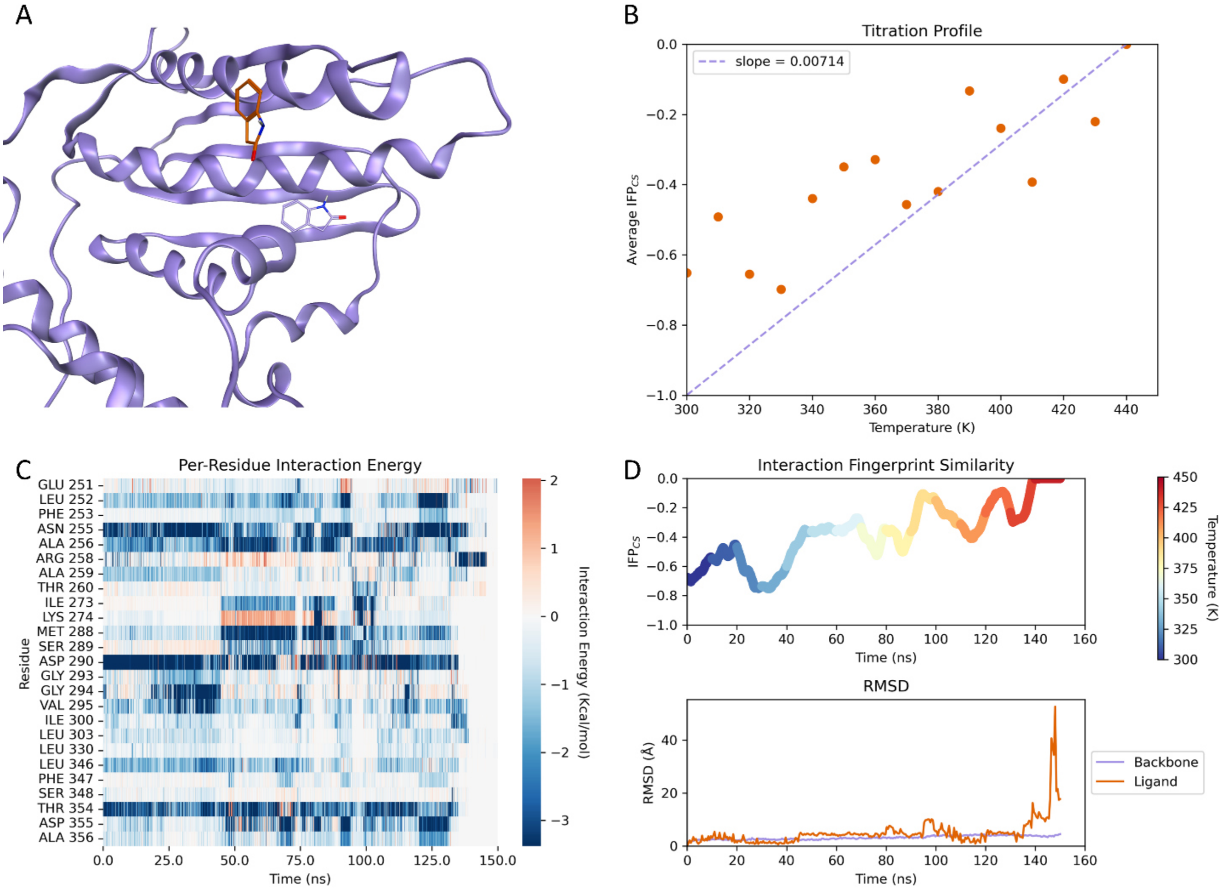

Figure S12. Analyses performed on a representative TTMD trajectory (**MD4**) for the docking pose of ligand **W6P** (PDB ID: **7EA0**) associated with the lowest RMSD to the reference (**pose 1**). A) superposition between the ligand conformation sampled in the last trajectory frame (orange) and the reference ligand binding mode (violet). B) titration profile: the average IFPcs value (adimensional units) for each "TTMD step" is reported as a function of the step temperature (K) in the form of orange dots. A violet straight line connects the start and the final point of the TTMD simulation, with the slope of the straight line being reported in the legend. C) time-dependent per-residue decomposition of the protein-ligand interaction energy, defined as a sum of the van der Waals and electrostatic contribution. The 25 most contacted residues alongside the TTMD trajectory are reported on the vertical axis, while the simulation time (ns) is reported on the horizontal axis. D) upper: time-dependent evolution of the IFPcs score; lower: time-dependent evolution of the RMSD for both the ligand (orange) and the protein backbone (violet) throughout the TTMD simulation. In both plots, the simulation time (ns) is reported on the horizontal axis, while the IFPcs (adimensional units) and RMSD (Å) value, respectively, are reported on the vertical axis.

Table S10. Principal chemical properties of the M^{pro} ligands utilized in this work. For each ligand, the IC_{50}, the molecular weight, the formal charge, the logP, and the number of hydrogen bond acceptors and donors are reported.

2D structure	PDB ID	LIG ID	IC_{50} [nM]	MW	Charge	logP	acc H	don H
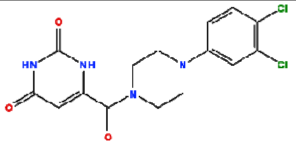	7LTJ	YD1	4200	369.21	0	2.43	3	2
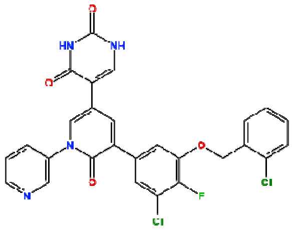	7M8P	YSJ	20	551.36	0	6.44	5	2
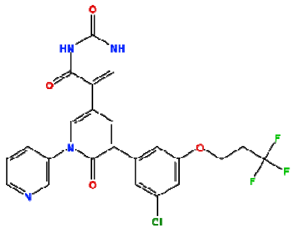	7M91	YU4	25	504.85	0	4.58	5	2

Table S11. Results of the TTMD post-docking refinement performed on docking poses generated for each investigated M^{pro} protein-ligand complex. For each pose, the representative replicate (MS value nearest to the average) is highlighted in violet.

PDB ID	Pose	MD1	MD2	MD3	MD4	MD5	Mean MS
**7LTJ** MS: 0.0066	1	0.0071	0.0083	**0.0077**	0.0083	0.0065	0.0077
	2	0.0100	0.0143	**0.0083**	0.0077	0.0031	0.0087
	3	0.0077	0.0059	0.0062	**0.0071**	0.0091	0.0070
	4	0.0083	0.0066	**0.0071**	0.0077	0.0067	0.0072
	5	**0.0077**	0.0083	0.0071	0.0071	0.0091	0.0077
**7M8P** MS: 0.0034	1	0.0026	0.0032	0.0033	0.0025	**0.0030**	0.0029
	2	0.0111	0.0125	0.0067	**0.0091**	0.0056	0.0090
	3	0.0062	0.0067	0.0063	0.0062	**0.0063**	0.0063
	4	0.0083	0.0111	**0.0062**	0.0059	0.0060	0.0068
	5	0.0200	0.0063	0.0062	0.0167	**0.0067**	0.0099
**7M91** MS: 0.0034	1	**0.0043**	0.0035	0.0067	0.0046	0.0029	0.0041
	2	0.0055	**0.0067**	0.0111	0.0066	**0.0067**	0.0067
	3	0.0077	**0.0067**	0.0071	0.0066	**0.0067**	0.0068
	4	**0.0048**	0.0047	0.0066	0.0125	0.0041	0.0054
	5	**0.0067**	0.0071	0.0045	0.0111	**0.0067**	0.0068

Table S12. Ligand RMSD (Å) sampled in the last frame of each TTMD simulation performed for the post-docking refinement of poses generated for each investigated M^{pro} protein-ligand complex.

PDB ID	Pose	MD1	MD2	MD3	MD4	MD5	Mean RMSD
**7LTJ**	1	80.29	55.29	45.80	45.25	9.90	48.78
	2	17.91	6.44	29.19	63.09	3.64	17.85
	3	45.21	8.34	9.09	28.51	77.72	27.60
	4	25.96	25.88	88.91	43.33	12.75	31.72
	5	44.80	7.74	45.65	24.26	63.12	38.24
**7M8P**	1	2.00	5.41	2.06	3.21	1.66	2.42
	2	26.60	32.05	44.11	16.88	7.22	25.18
	3	9.46	38.18	7.49	9.71	7.08	8.89
	4	41.17	53.71	10.42	8.68	7.35	20.09
	5	8.66	7.21	9.52	25.43	33.18	14.54
**7M91**	1	2.22	2.12	4.52	2.22	3.22	2.55
	2	6.30	24.07	65.63	10.40	34.62	23.03
	3	15.18	30.45	35.79	11.26	31.01	25.55
	4	4.43	3.01	30.11	62.55	4.34	12.96
	5	23.54	35.40	6.05	21.94	38.09	26.96

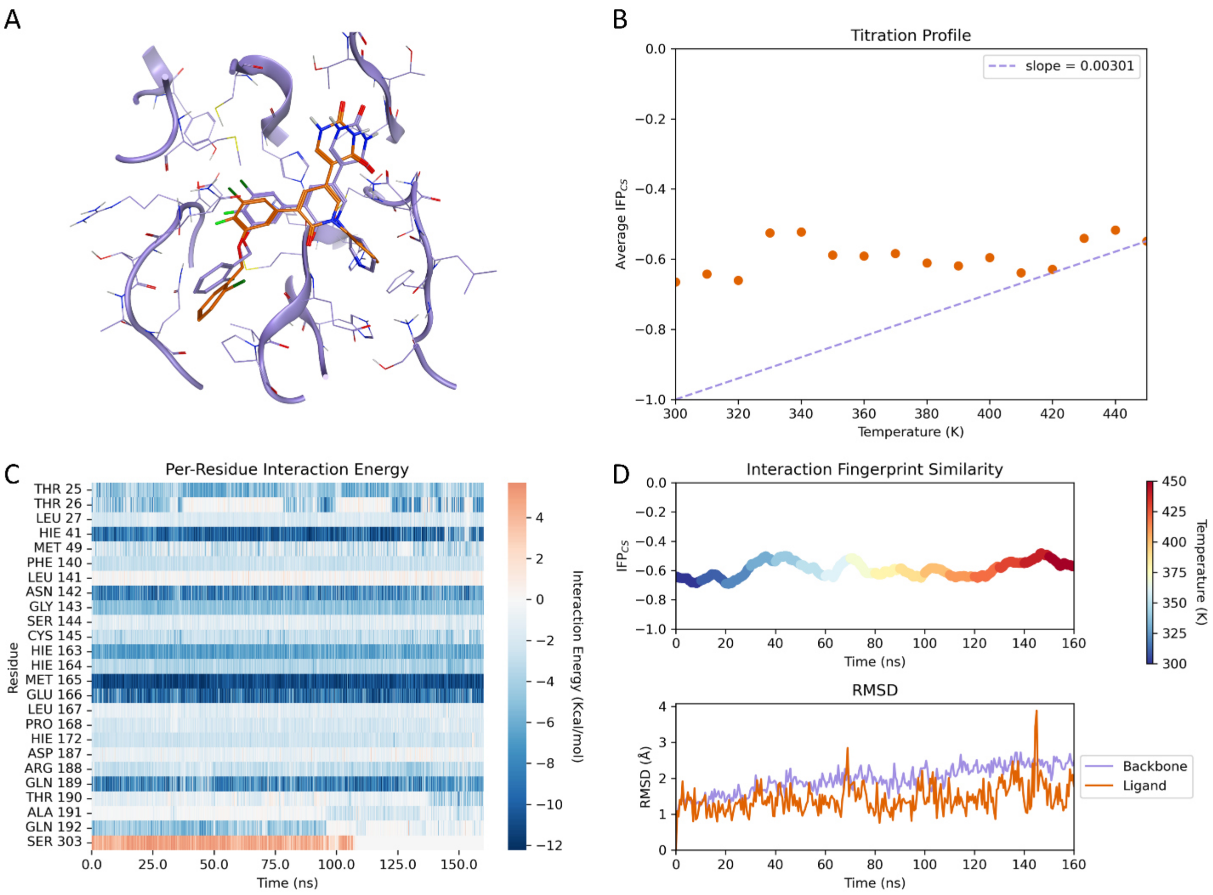

Figure S13. Analyses performed on a representative TTMD trajectory (**MD5**) for the docking pose of ligand **YSJ** (PDB ID: **7M8P**) associated with the lowest MS score (**pose 1**). A) superposition between the ligand conformation sampled in the last trajectory frame (orange) and the reference ligand binding mode (violet). B) titration profile: the average IFPcs value (adimensional units) for each "TTMD step" is reported as a function of the step temperature (K) in the form of orange dots. A violet straight line connects the start and the final point of the TTMD simulation, with the slope of the straight line being reported in the legend. C) time-dependent per-residue decomposition of the protein-ligand interaction energy, defined as a sum of the van der Waals and electrostatic contribution. The 25 most contacted residues alongside the TTMD trajectory are reported on the vertical axis, while the simulation time (ns) is reported on the horizontal axis. D) upper: time-dependent evolution of the IFPcs score; lower: time-dependent evolution of the RMSD for both the ligand (orange) and the protein backbone (violet) throughout the TTMD simulation. In both plots, the simulation time (ns) is reported on the horizontal axis, while the IFPcs (adimensional units) and RMSD (Å) value, respectively, are reported on the vertical axis.

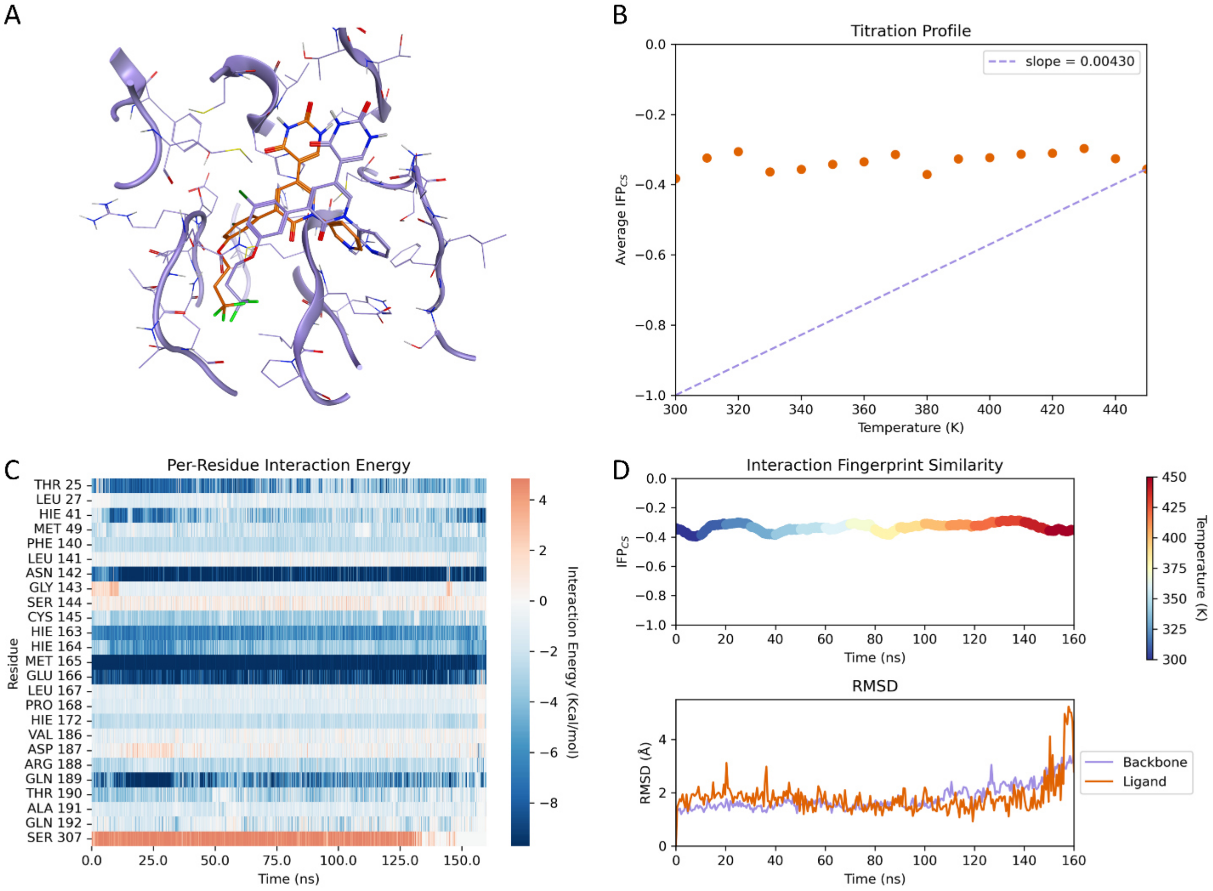

Figure S14. Analyses performed on a representative TTMD trajectory (**MD1**) for the docking pose of ligand **YU4** (PDB ID: **7M91**) associated with the lowest MS score (**pose 1**). A) superposition between the ligand conformation sampled in the last trajectory frame (orange) and the reference ligand binding mode (violet). B) titration profile: the average IFPcs value (adimensional units) for each "TTMD step" is reported as a function of the step temperature (K) in the form of orange dots. A violet straight line connects the start and the final point of the TTMD simulation, with the slope of the straight line being reported in the legend. C) time-dependent per-residue decomposition of the protein-ligand interaction energy, defined as a sum of the van der Waals and electrostatic contribution. The 25 most contacted residues alongside the TTMD trajectory are reported on the vertical axis, while the simulation time (ns) is reported on the horizontal axis. D) upper: time-dependent evolution of the IFPcs score; lower: time-dependent evolution of the RMSD for both the ligand (orange) and the protein backbone (violet) throughout the TTMD simulation. In both plots, the simulation time (ns) is reported on the horizontal axis, while the IFPcs (adimensional units) and RMSD (Å) value, respectively, are reported on the vertical axis.

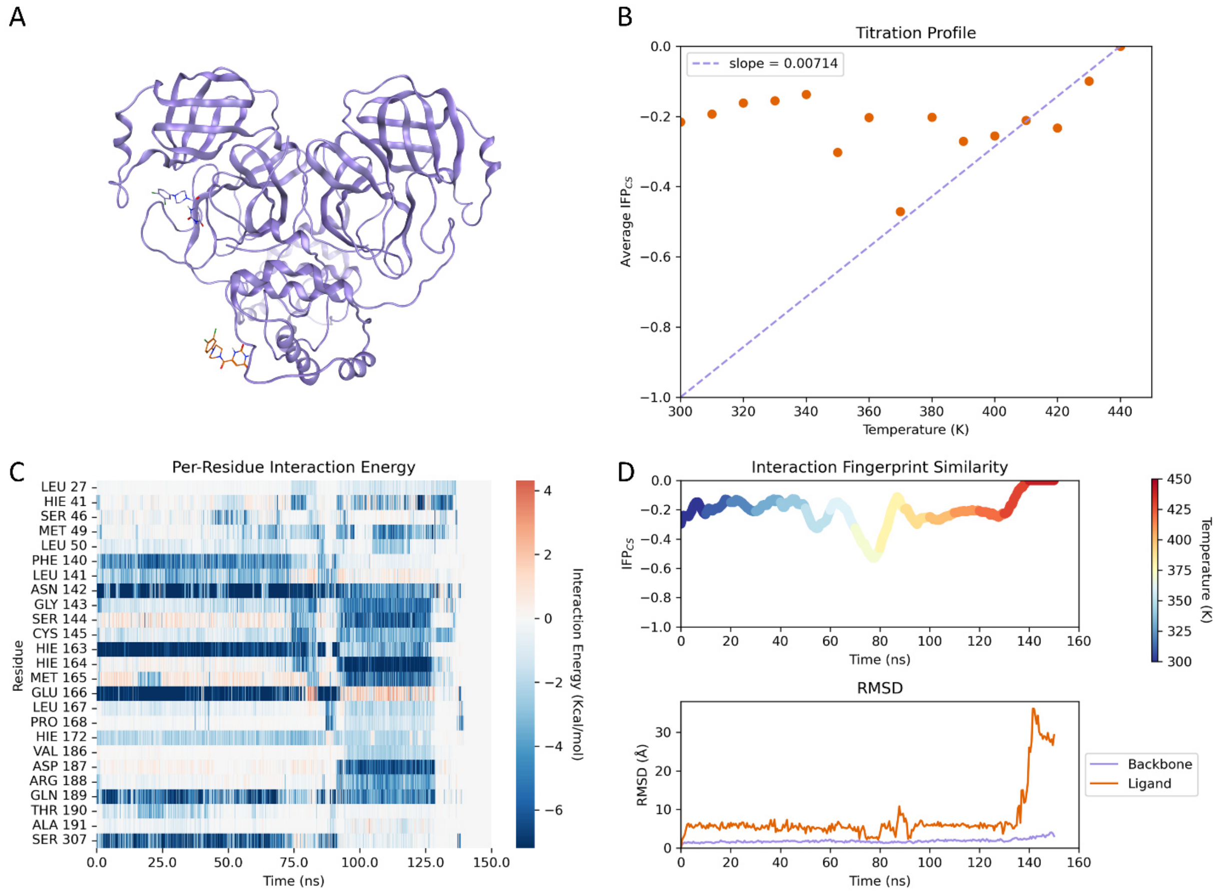

Figure S15. Analyses performed on a representative TTMD trajectory (**MD4**) for the docking pose of ligand **YD1** (PDB ID: **7LTJ**) associated with the lowest MS score (**pose 3**). A) superposition between the ligand conformation sampled in the last trajectory frame (orange) and the reference ligand binding mode (violet). B) titration profile: the average IFPcs value (adimensional units) for each "TTMD step" is reported as a function of the step temperature (K) in the form of orange dots. A violet straight line connects the start and the final point of the TTMD simulation, with the slope of the straight line being reported in the legend. C) time-dependent per-residue decomposition of the protein-ligand interaction energy, defined as a sum of the van der Waals and electrostatic contribution. The 25 most contacted residues alongside the TTMD trajectory are reported on the vertical axis, while the simulation time (ns) is reported on the horizontal axis. D) upper: time-dependent evolution of the IFPcs score; lower: time-dependent evolution of the RMSD for both the ligand (orange) and the protein backbone (violet) throughout the TTMD simulation. In both plots, the simulation time (ns) is reported on the horizontal axis, while the IFPcs (adimensional units) and RMSD (Å) value, respectively, are reported on the vertical axis.

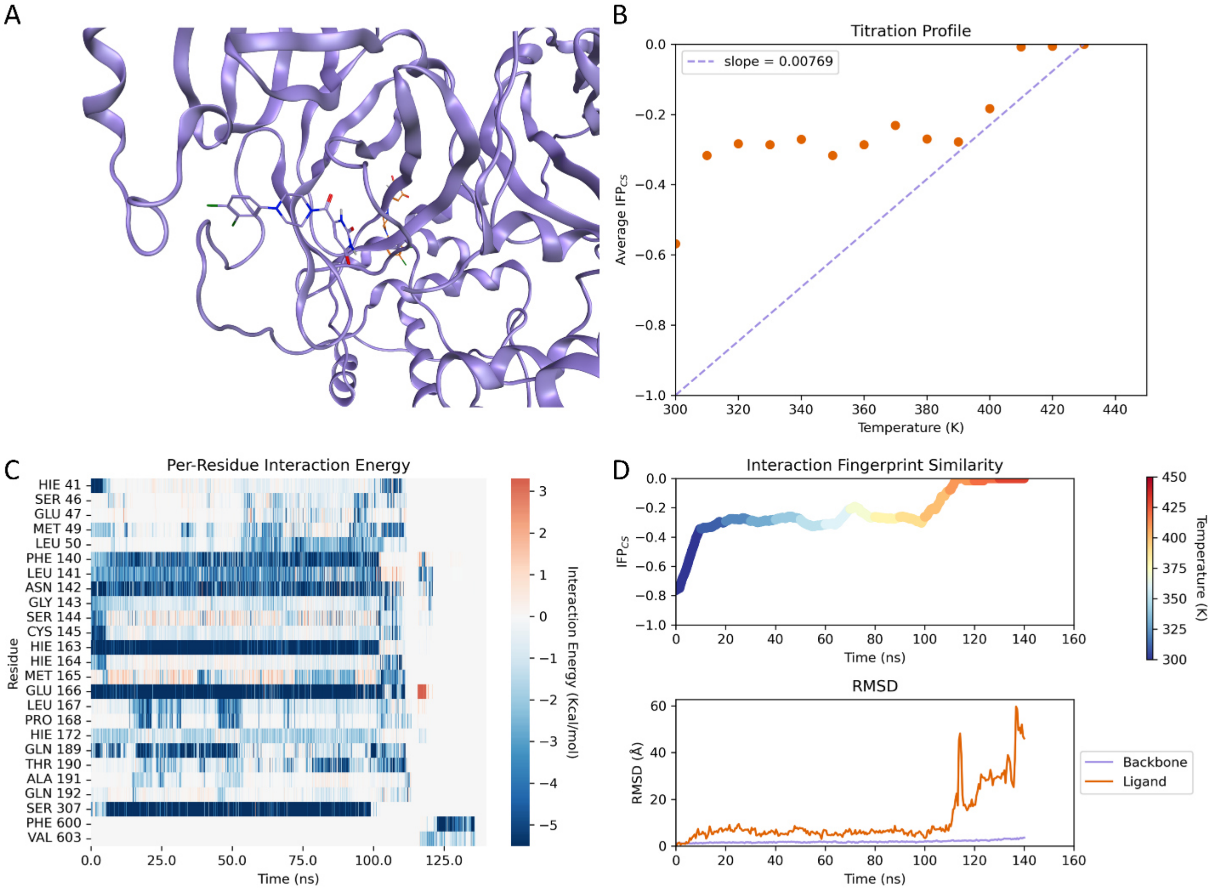

Figure S16. Analyses performed on a representative TTMD trajectory (**MD3**) for the docking pose of ligand **YD1** (PDB ID: **7LTJ**) associated with the lowest RMSD to the reference (**pose 1**). A) superposition between the ligand conformation sampled in the last trajectory frame (orange) and the reference ligand binding mode (violet). B) titration profile: the average IFPcs value (adimensional units) for each "TTMD step" is reported as a function of the step temperature (K) in the form of orange dots. A violet straight line connects the start and the final point of the TTMD simulation, with the slope of the straight line being reported in the legend. C) time-dependent per-residue decomposition of the protein-ligand interaction energy, defined as a sum of the van der Waals and electrostatic contribution. The 25 most contacted residues alongside the TTMD trajectory are reported on the vertical axis, while the simulation time (ns) is reported on the horizontal axis. D) upper: time-dependent evolution of the IFPcs score; lower: time-dependent evolution of the RMSD for both the ligand (orange) and the protein backbone (violet) throughout the TTMD simulation. In both plots, the simulation time (ns) is reported on the horizontal axis, while the IFPcs (adimensional units) and RMSD (Å) value, respectively, are reported on the vertical axis.

Table S13. Principal statistics about each simulated system. System dimension, total number of atoms, water molecules, sodium and chlorine ions, and average simulation time are reported.

PDB ID	System dimension [Å]			N° atoms	N° H_2O	N° Na^+	N° Cl^-	Speed [ns/day]	
	X	Y	Z					Nvidia 1080Ti	Nvidia 2080Ti
**3UZP**	89.18	93.87	91.00	62441	19150	49	61	124	209
**5IH6**	88.30	88.48	92.91	60101	18412	47	59		
**5MQV**	88.59	97.41	92.05	65587	20207	53	65		
**3PE1**	101.58	87.18	92.05	67679	20677	60	60	108	182
**3PE2**	100.86	87.92	91.28	67213	20522	60	59		
**6HOU**	100.86	89.64	93.52	70656	21669	63	63		
**4V25**	86.98	100.47	100.10	72856	22314	68	62	100	172
**5M4M**	84.95	100.47	99.88	71070	21719	67	60		
**7EA0**	96.50	89.64	99.88	72347	22161	67	62		
**7LTJ**	109.10	101.41	96.79	89755	26733	81	73	77	142
**7M8P**	111.69	100.47	109.78	104646	31708	96	89		
**7M91**	97.43	100.10	119.73	98912	29783	89	83		
